# Belowground Ecology of Scarabs Feeding on Grass Roots: Current Knowledge and Future Directions for Management in Australasia

**DOI:** 10.3389/fpls.2016.00321

**Published:** 2016-03-22

**Authors:** Adam Frew, Kirk Barnett, Uffe N. Nielsen, Markus Riegler, Scott N. Johnson

**Affiliations:** Hawkesbury Institute for the Environment, Western Sydney UniversityRichmond, NSW, Australia

**Keywords:** *Anoplognathus*, belowground herbivory, *Cyclocephala signaticollis*, *Dermolepida albohirtum*, *Heteronychus arator*, pasture, pest management, *Sericesthis nigrolineata*

## Abstract

Many scarab beetles spend the majority of their lives belowground as larvae, feeding on grass roots. Many of these larvae are significant pests, causing damage to crops and grasslands. Damage by larvae of the greyback cane beetle (*Dermolepida albohirtum*), for example, can cause financial losses of up to AU$40 million annually to the Australian sugarcane industry. We review the ecology of some scarab larvae in Australasia, focusing on three subfamilies; Dynastinae, Rutelinae, and Melolonthinae, containing key pest species. Although considerable research on the control of some scarab pests has been carried out in Australasia, for some species, the basic biology and ecology remains largely unexplored. We synthesize what is known about these scarab larvae and outline key knowledge gaps to highlight future research directions with a view to improve pest management. We do this by presenting an overview of the scarab larval host plants and feeding behavior; the impacts of abiotic (temperature, moisture, and fertilization) and biotic (pathogens, natural enemies, and microbial symbionts) factors on scarab larvae and conclude with how abiotic and biotic factors can be applied in agriculture for improved pest management, suggesting future research directions. Several host plant microbial symbionts, such as arbuscular mycorrhizal fungi and endophytes, can improve plant tolerance to scarabs and reduce larval performance, which have shown promise for use in pest management. In addition to this, several microbial scarab pathogens have been isolated for commercial use in pest management with particularly promising results. The entomopathogenic fungus *Metarhizium anisopliae* caused a 50% reduction in cane beetle larvae while natural enemies such as entomopathogenic nematodes have also shown potential as a biocontrol. Key abiotic factors, such as soil water, play an important role in affecting both scarab larvae and these control agents and should therefore feature in future multi-factorial experiments. Continued research should focus on filling knowledge gaps including host plant preferences, attractive trap crops, and naturally occurring pathogens that are locally adapted, to achieve high efficacy in the field.

## Introduction

Worldwide there are over 31,000 species of scarab beetles (Coleoptera: Scarabaeidae; Jameson, 2015) and within Australia alone there are well over 2,200 described species (Hangay and Zborowski, 2010). These scarabs can be found across tropical, subtropical and temperate regions of Australia and New Zealand in a broad range of ecosystem types including agroecosystems (Allsopp, 1999). Many scarabs have become destructive pests of grasslands as root-feeders (Potter and Braman, 1991). There are also instances where introduced plant species have become the preferred host to a number of native scarabs such as greyback cane beetle larvae (*Dermolepida albohirtum* Waterhouse, subfamily: Melolonthinae) feeding on sugarcane (*Saccharum* sp.). Moreover, the problem of such species becoming pests has been exacerbated by agriculture (Robertson et al., 1995), such as large-scale transition of grassland into arable crop production, or of forests and woodlands into pastures. Crop losses due to scarab larval damage for sugarcane in Australia alone can result in losses up to AU$40 million annually (Chandler, 2002). Historically, this problem has been addressed by using chemical pesticides, which can have serious collateral effects on non-target organisms and the environment (Jackson and Klein, 2006). As such, alternative management strategies are being continually investigated (Goldson et al., 2015).

Understanding the biology and behavior of scarab larvae, including their interactions with host plants and the soil environment (or rhizosphere) is an essential component to enabling effective management and control, both in Australia and at a global scale. There are numerous studies on these larvae within Australasia, some of which have elucidated core biology, behavior and even responses to future environment such as climate change (Johnson et al., 2014). However, for many scarab species this work was carried out some time ago, while for others the majority of their ecology has yet to be described. This is partly due to their soil-dwelling habit which has made culturing and experimentation particularly challenging. It is therefore timely to synthesize the fragmented information available on this group of root-feeding pests in Australasia. In this review we identify where knowledge is lacking, highlight promising research avenues into pest management, to suggest where continued research should be focused. In particular, this review focuses on belowground influences which impact larval development and survival. Edaphic variables such as soil moisture and temperature alongside biotic interactions with microbiota both in the soil and with host plants show most promise for improved pest management.

We concentrate on three subfamilies belonging to the family Scarabaeidae: Dynastinae (e.g., African black beetle *Heteronychus arator* Fabricius and Argentine scarab *Cyclocephala signaticollis* Burmeister), Rutelinae (e.g., Christmas beetles *Anoplognathus* sp. Leach) and Melolonthinae (e.g., dusky pasture scarab *Sericesthis nigrolineata* Boisduval and greyback cane beetle *D. albohirtum*). Within these subfamilies we focus on the key pest species/genera examples mentioned, while including any relevant information from other species within the subfamilies. The redheaded cockchafer, *Adoryphorus couloni* Burmeister (subfamily: Dynastinae) is also a significant pasture pest within Australia and was comprehensively reviewed recently (Berg et al., 2014). Hence, we do not include this species within the review. Within the three subfamilies we specifically focus on:

1. Host plants and feeding behavior
2. Abiotic soil factors (temperature, moisture, and fertilization)
3. Biotic soil factors (pathogens, natural enemies, and symbionts)
4. Applied perspectives
5. Directions for future research

## Host Plants and Feeding Behavior

**FIGURE 1:**
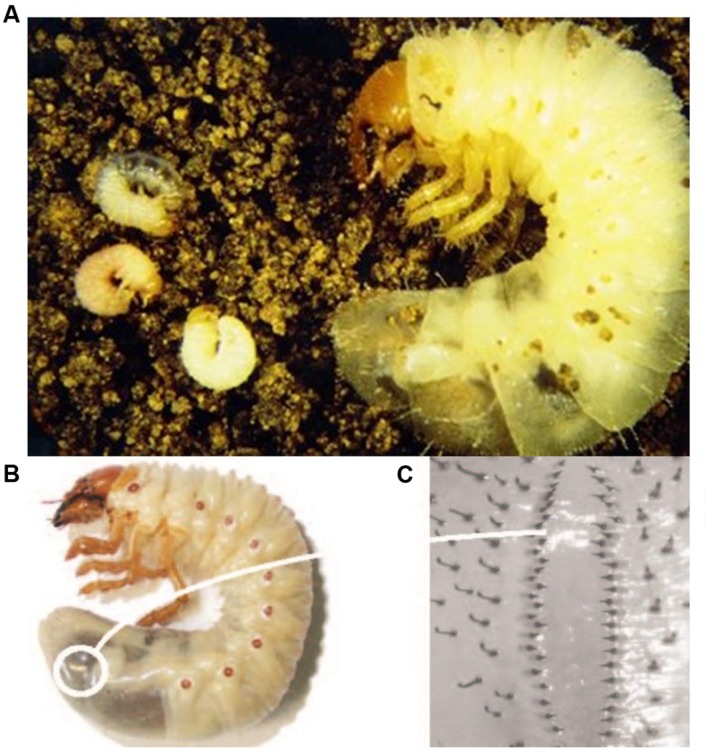
**Scarab larvae: (A) African black beetle larvae *Heteronychus arator*, (B) greyback cane beetle larva *Dermolepida albohirtum*, (C) close-up of hair pattern (raster) used to identify greyback cane beetle larvae.** Images supplied by Western Australian Department of Agriculture and Food (African black beetle) and Sugar Research Australia (greyback cane beetle larva).

**FIGURE 2:**
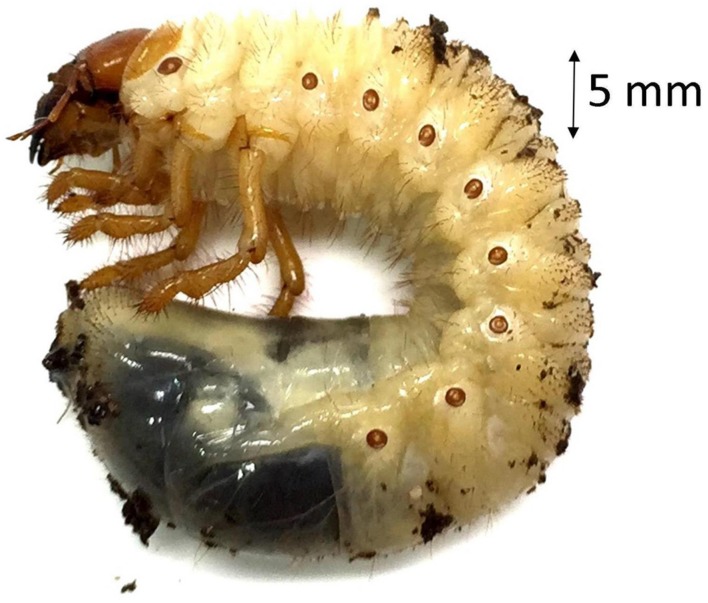
**Third instar larva of the greyback cane beetle (*D. albohirtum*).** Image supplied by Adam Frew.

**FIGURE 3:**
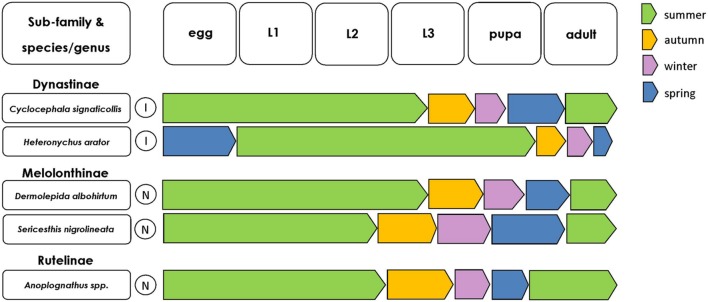
**Seasonal occurrence of scarab life stages for each of the key scarab pest species.** Such information can help to design life-stage specific, targeted pest control programs. Color of arrows indicates the season in which each scarab life stage typically occurs (within Australia and New Zealand). Circles with I indicate species invasive to Australasia, circles with N indicate species native to Australasia.

While the majority of scarabs are grass root-feeders in their larval stages (**Figures 1** and **2**; Goodyer and Nicholas, 2007), some larvae feed on organic matter in the soil litter (Jackson and Klein, 2006). For some pest scarab species, feeding ecology has been documented relatively well. Across the subfamilies discussed here the most damaging and voracious feeding occurs during the third instar, therefore the timing of development of pest scarab larvae is important to consider from a pest management perspective (**Figure 3**). Indeed, the ability of all scarab larvae to locate suitable hosts is equally as important as the nutritional value of the host plant. Carbon dioxide emissions by the host plant is an important root exudate that plays a role in host plant location by root herbivores (Johnson and Gregory, 2006); however, other volatile root exudates are clearly critical in host plant location by scarab larvae (Eilers et al., 2012). The topic of host plant location by root-feeders was reviewed by Johnson and Gregory (2006) and revised by Johnson and Nielsen (2012), and we will not discuss this in detail here. Here we will present what is known regarding the feeding behavior of some of the key species from within Dynastinae, Rutelinae, and Melolonthinae.

### Dynastinae

The African black beetle has been described as a sporadic pest of pastures and crops across New Zealand and Australia (Matthiessen and Ridsdill-Smith, 1991). Plant species composition influences the distribution of the African black beetle across the landscape (King and Kain, 1974; King et al., 1982). The larvae seem to have reduced performance on species such as *Medicago sativa* (King et al., 1975) and tend to avoid feeding on *Trifolium repens* (Sutherland and Greenfield, 1978), which is due, at least in part, to the feeding deterrents medicarpin and vestitol present in the roots (Russell et al., 1982). That said, larvae will eat *T. repens* roots if given no other choice (King et al., 1981c). Despite this, *T. repens* is a common food source for other scarab larvae such as *Costelytra zealandica* White (subfamily: Melolonthinae; King et al., 1981a; Russell et al., 1982; Prestidge et al., 1985).

By contrast, the grasses *Lolium perenne* and *Paspalum dilatatum* have been shown to be a preferred food choice of pasture grass species (King, 1977; King et al., 1981a). King (1977) found that African black beetle larval mass gain was greater on *L. perenne* when compared with *T. repens* and *Lotus pedunculatus*, but also that organic matter in the soil stimulated this feeding and increased weight gain. The organic content of the soil acting as a feeding stimulant has therefore been suggested as having implications for damage in soil with high peat content (Bell et al., 2011). Indeed the African black beetle is a significant pest of *L. perenne* pastures, both as larvae and adults, feeding on below- and aboveground portions of the plant, respectively (Popay and Bonos, 2008). The endophytic fungus *Neotyphodium lolii*, forms a mutualistic relationship with *L. perenne* (Raman et al., 2012). Feeding by adult African black beetles is well documented to be deterred by *N. lolii* infected *L. perenne* (Popay and Baltus, 2001), which has been attributed to the presence of alkaloids (Thom et al., 2014). More recently, Qawasmeh et al. (2015) found that different strains of *N. lolii* had an impact on the aboveground volatile profile of *L. perenne* and the attractiveness of this host plant to adult African black beetles.

The majority of research into endophyte (**Table 1**) induced protection has focused on aboveground herbivores (Popay and Baltus, 2001). One study on a specific *N. lolii* strain noted that the African black beetle larvae were observed to have a reduced occurrence in *N. lolii* infected grasses (Hume et al., 2007). More recently, another study has found changes in the root volatile profile in response to *Neotyphodium uncinatum* infection and found decreased attraction to *C. zealandica* larvae belowground (Rostás et al., 2015).

**Table 1:** Glossary of terms.

Term	Explanation
Endophyte	Bacterium or fungus which lives within a plant; endophyte infected ryegrass deter feeding by African black beetles.
Entomopathogenic fungus	Fungus which is a parasite to insects, often killing them; particular fungi have been used as part of pest management of scarab larvae.
Entomopathogenic nematode	Nematodes (thread worms) which kill insects via the bacteria they harbor inside them; some species have been used as part of pest management of scarab larvae.
Endosymbiotic bacteria	Bacteria living within another organism; found in the hindguts of scarab larvae, aiding digestion of plant material.

Considering damage can be significant, more research focusing on the efficacy of *N. lolii* strains in deterring African black beetle larvae would be the logical next steps. In the field, replacing turfgrass or pasture with *N. lolii* infected *L. perenne* could convey protection against African black beetle adults at the very least, perhaps reducing oviposition, and indeed may deter all alkaloid sensitive insect herbivores (see ‘Applied perspectives’ section).

The feeding behavior of Argentine scarab larvae has not received significant attention in the literature despite its pest status on turf and pastures (Carne, 1957a). Within Argentina, the larvae are known as pests particularly of potato crops (Berón and Diaz, 2005), but are known to feed on roots of flax, lucerne, sunflower, and carrot crops as well (Mondito et al., 1997). In Australia, however, the larvae feed mainly on grass roots. Carne (1957a) noted that the larvae were found in the greatest numbers in grasslands with *Cynodon dactylon* and *P. dilatatum*. It was also noted that this scarab could successfully develop on a diet composed solely of decomposing organic matter; however, the abundance found in pastures indicates some of their nutrient requirements are derived from grass roots. It is evident the Argentine scarab larvae feed on both organic matter and actively on grass roots but other than a few studies no other feeding behavior investigation has been carried out on the Argentine scarab in Australian grasslands. The lack of context specific studies on the larval feeding preferences of this scarab species, alongside the efficacy of management practices, calls for initial host preference studies to be conducted before any control initiatives can effectively be researched and applied.

### Rutelinae

The feeding behavior of adult *Anoplognathus* spp., which consume the leaves of eucalypts, is addressed well within the literature (Carne et al., 1974; Edwards et al., 1993; Steinbauer and Wanjura, 2002; Johns et al., 2004; Steinbauer and Weir, 2007), in contrast to the information on larval feeding behavior, which is relatively scarce.

*Anoplognathus* larvae are known to feed on organic matter in the soil, grass roots, and crop roots (Carne, 1957b; Sallam et al., 2011). Some species within the genus, such as *Anoplognathus montanus*, will commonly feed on rotting organic material such as timber, but will also feed on the finer roots of eucalypts (Carne, 1957b). Carne et al. (1974) stated that larvae of *Anoplognathus* feed primarily on organic matter in the soil and tend not to seek out plant roots. While Davidson and Roberts (1968a) confirmed this, they nonetheless stated that the organic matter they feed on is composed mainly of plant roots. Here, they also found that when Christmas beetle larvae fed on the grass *Phalaris tuberosa* and *T. repens*, larvae often failed to reach pupation, which could be due to secondary metabolites in the plant. In a further study that year, it was found that Christmas beetle larvae avoided feeding on *T. repens* altogether (Davidson and Roberts, 1968b), a behavior also exhibited by African black beetle larvae.

The larvae of *Anoplognathus* spp. have been reported as pests of sugarcane, although only when numbers are high (Samson et al., 2013). Significant damage to pastures by Christmas beetle larvae is well known, particularly by the third instar (Urquhart, 1995). Feeding populations of larvae can be influenced by aboveground herbivores. A study by Roberts and Morton (1985) investigated the effects of grazing pressure on the biomass of *Anoplognathus* sp. larvae, and found that larval abundance peaked under low to intermediate grazing pressure. Therefore, low pasture damage by larvae may be exacerbated by moderate grazing of livestock aboveground.

### Melolonthinae

**FIGURE 4:**
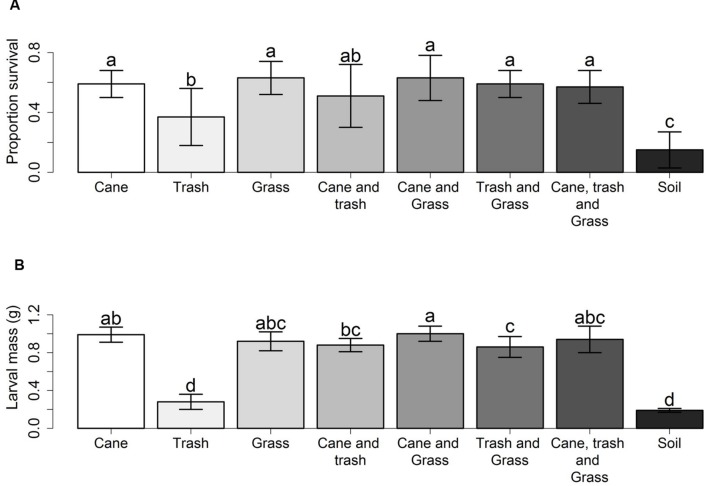
**Survivorship and mass of early instar larvae of *D. albohirtum*. (A)** Mean proportion survival (±SD), and **(B)** mean larval mass in grams (±SD), of larvae after 4 weeks in bins with food of either sugarcane, Guinea grass, cane trash, combinations of two or three of these, or none of these. Different letters indicate significant effects of treatments. Adapted from Logan and Kettle (2002).

The greyback cane beetle is a long standing pest within sugarcane and the larvae can cause devastating damage to crops (Chandler, 2002). Initial uncertainties regarding the feeding of mainly organic material in the soil (Illingworth and Dodd, 1921) have been resolved as there is compelling evidence for grass roots as the main resource (Sallam, 2011). Root feeding was shown by Logan and Kettle (2002) who investigated the effect of food type on the survival and development of first instar greyback cane beetle larvae. Larval survival and development was highest in treatments with grass seedlings and lowest in soil alone. This result was confirmed by a second experiment using sugarcane, Guinea grass (*Panicum maximum*), cane trash (mulch), and a soil only environment, where larval survival and mass was lowest in the soil only treatment and highest when cane or grass were available (**Figure 4**).

In Australia, cane beetles are the major pests to the sugar industry (McLeod et al., 1999; Horsfield et al., 2008) and as a result there have been several studies into pest management and environmental conditions that may impact on larval induced damage to sugarcane (Robertson et al., 1995; Robertson and Walker, 2001; Chandler, 2002). Coupled with the development of pest management strategies, Allsopp (1991) investigated feeding stimulants of greyback cane beetle larvae, which could be used to enhance the efficacy of larval baits. Larvae showed a strong feeding response to fructose and sucrose. Both sucrose and fructose, along with glucose, are the most abundant sugars found in sugarcane, and are both at higher concentrations in the lower stem of sugarcane compared with the roots (Irvine, 1977).

Estimates of population size and density within sugarcane fields vary from three or four larvae per cane plant (Ward and Robertson, 1999) to numbers of 15 per plant, or more (Jarvis, 1933; Sallam, 2011). Some Melolonthinae larvae have shown specific soil type preferences. A study by Cherry and Allsopp (1991) found distinct soil type preferences between different species, with some larval populations of some species positively correlated with clay and silt, and negatively with sand content, while other species showed opposing correlations. For yet other species, such as the greyback cane beetle, soil type has little influence on the distribution (Robertson and Walker, 2001). Overall there is no ‘one soil type fits all’ for scarab species as studies have shown species specific preferences (Gordon and Anderson, 1981; Cherry and Hall, 1986).

Studies conducted into the feeding behavior of dusky pasture scarab larvae have focused on climatic and abiotic influences rather than host preference. The larvae can feed and survive in soil in the absence of plant roots (Ridsdill-Smith, 1975; Smith and Porter, 1980), however, it is not clear if they are able to develop into adults on soil organic matter alone. The feeding behavior, and relative consumption of food is largely influenced by temperature (Davidson et al., 1972b; Ridsdill-Smith et al., 1975; Cairns, 1978) and under field conditions there is often a seasonal pattern of larval feeding as a result of local temperatures. Ridsdill-Smith (1975) carried out an investigation into the feeding behavior of dusky pasture scarab larvae using slices of carrot under different temperatures. It was found that the larval consumption of food peaked at 30°C while, interestingly, the efficiency of conversion of ingested food (which accounts for larval growth and the mass of food consumed) peaked at a temperature of 14°C. To build upon this, a follow-up study utilizing larvae that had been fed on living roots and a variety of food sources was conducted by Ridsdill-Smith (1977). It found that the feeding of dusky pasture scarab larvae declined when the population densities were high, although this was likely a result of a lack of young living roots. This was confirmed by Ridsdill-Smith and Roberts (1976), who also showed that larval growth reduced as density increased, which was also likely to be due to a limited food supply. The study also suggested that the larvae preferred to feed on younger roots.

One recent study by Johnson et al. (2014) provided evidence of compensatory feeding by the dusky pasture scarab larvae under elevated atmospheric CO_2_ (eCO_2_) on *Microlaena stipoides*, a C_3_ grass. Despite this increased feeding, the performance of the dusky pasture scarab was much lower under these eCO_2_ conditions, which was likely due to a reduction in the root nitrogen concentrations. Interestingly, under ambient CO_2_, larvae consumed 48% more material from *M. stipoides* than from *Cymbopogon refractus*, a C_4_ grass. Generally, C_3_ grasses are thought to be more susceptible to herbivory than C_4_ grasses (Caswell et al., 1973). More studies of this type are necessary to elucidate the relationship between scarabs and their host plants, particularly when considering changes in feeding behaviors as a result of climate change. It can be concluded from these studies that the feeding behavior of the dusky pasture scarab larvae is strongly influenced by abiotic factors such as temperature and, indirectly, atmospheric CO_2_. As such, future research should investigate host plant preferences alongside abiotic and biotic interactions, including changes in atmospheric CO_2_ concentrations.

## Abiotic Soil Factors

Abiotic factors have been seen to have a strong influence on insect pests of Australasia (Powell et al., 2003). All root-feeding insects respond directly to their immediate physical and chemical environment (Barnett and Johnson, 2013). Here, we review some significant abiotic factors impacting on scarab larvae: temperature, moisture, and fertilization. We focus on species within Dynastinae, Rutelinae, and Melolonthinae found in Australasia. We also draw on studies of other species within these subfamilies outside Australasia to indicate the general impact of abiotic rhizospheric factors on scarab larvae. These factors are considered with a view to highlight where agricultural practices could be modified to reduce damage by scarab larvae (discussed in more detail in ‘Applied perspectives’ section).

### Temperature

The temperature of the soil can impact significantly on scarabs, particularly in the egg and early larval stages. For example, temperature has been seen to have an impact on population fluctuations of the African black beetle (East et al., 1981; King et al., 1981b). Despite this importance, few studies have focused on the temperature preferences for oviposition by scarab females.

Regarding larval stages, a single exposure of 35°C for 24 h has been shown to kill 100% of first instar larvae of *Anoplognathus* spp. and the dusky pasture scarab, while around 62% survive when exposure to such temperatures is only for 12 h (Hassan and Hilditch, 1976). Within the same study, second instar larvae showed a higher tolerance for high temperatures, for example at 37.5°C, 73% of first instar larvae died while only 40% of second instar died. Regarding the lower temperature threshold it is generally understood that at low temperatures (below 16°C) scarab eggs will take longer to hatch and larvae will take longer to develop (Davidson et al., 1972b). This relationship between temperature and development was investigated in greyback cane beetle pupae (Logan and Kettle, 2007), where the minimum and maximum time for pupal development was found to be 26 days at 30°C and 75 days at 18°C, respectively. The low temperature threshold, at and below which no development occurs was 12°C. There have several studies showing the influence of temperature on the growth and development of the dusky pasture scarab (Davidson et al., 1972b; Ridsdill-Smith, 1975; Ridsdill-Smith et al., 1975; Cairns, 1978). The relative growth rate of these larvae was found to have lower and upper temperature limits of 5°C and 32°C, respectively, with optimum growth occurring around 17.5°C (Ridsdill-Smith et al., 1975). One study on *Rhizotragus majalis* Razoumowsky (subfamily: Melolonthinae), indicated that later instar larvae have much greater mobility and therefore older scarab larvae are likely to be less susceptible to temperature stress through avoidance behavior (Villani and Nyrop, 1991). This was confirmed by Zhang et al. (2003) who confirmed higher mobility in second and third instars by monitoring their acoustic sounds, which also increased with soil temperature, while below 9°C sound production fell to a minimum. Overall, temperature plays an important role in the survival, and the rate of development of scarab larvae. Generally, larval growth rate increases with temperature, where upper limits tend to be between 35 and 40°C, and as temperatures drop to 16°C or below, development is significantly reduced. First instar larvae tend to be the most sensitive to temperatures stress, while scarab eggs and later instar larvae are more tolerant.

These larval responses to temperature indicate how significant climate can be to larval populations. Indeed, high temperatures at a particular time of development can have particularly large impacts on greyback cane beetle populations. Horsfield et al. (2008) analyzed larval damage records and climatic averages from 1989 to 2003 and showed that prolonged hot and dry conditions during the late spring can limit population numbers by impacting on emergence, as well as synchrony of emergence with feeding, mating and egg laying. Conversely, milder and wetter spring season can promote adult emergence and the ability of the adults to successfully feed, mate and lay eggs. This would directly impact on successive larval populations and therefore damage to cane the following year.

### Moisture

Soil moisture is often referred to as the most important property that affects the development and survival of scarab larvae belowground (Brown and Gange, 1990; Barnett and Johnson, 2013). Indeed, eggs of many scarab species must absorb water before hatching (Potter, 1983), and hence the availability of water in the soil can be critical to scarab population dynamics. Soil moisture is also the factor best examined in the literature with regards to female oviposition in scarabs (Potter, 1983; Cherry et al., 1990; Allsopp et al., 1992; Logan, 2007). Several studies have shown different optimal soil moisture conditions for maximum oviposition. Some Melolonthinae scarabs are known to oviposit in soils around field capacity (-74 kPa; Logan, 2007), while others within the same subfamily prefer a range between field capacity and dry soil near wilting point (-1500 kPa; Logan, 2007). Ward and Rogers (2007) carried out a study on soil moisture ovipositional preferences in four Melolonthinae scarabs found in Australia, including the greyback cane beetle. It was concluded that those species adapted to the semi-arid tropics, where rainfall is unreliable, have little or no preferences observed beyond a reduction in oviposition in very dry soil (-1500 kPa). However, in subtropical and temperate (with less seasonal rainfall) adapted species there were clear preferences for drier soils (-1000 kPa). This suggests that the climates in which key/target pest species have originated and are adapted to, must be considered in attempts to manage populations. It also indicates that for those tropically adapted species, moisture control as a form of pest management may not be the way forward, as their ovipositional preferences are likely to be driven by factors other than soil moisture.

Moisture content of the soil can directly impact on scarab larvae populations. African black beetle populations, for example, have been shown to be suppressed in regions with early summer rainfall (Matthiessen and Ridsdill-Smith, 1991), as first instar larvae are more moisture sensitive than egg stage or later instars (King, 1979; King et al., 1981b). In periods of seasonal drought, the larval populations are no longer suppressed by the normally high moisture content, resulting in damaging outbreaks (Matthiessen and Ridsdill-Smith, 1991). Whether these population responses would be the same in different soils is uncertain. Matthiessen (1999) showed that soil type had a significant impact on African black beetle larval survival, and that this factor interacted with soil moisture, where larval survival was higher under regular watering treatments compared with no watering, but only in some soil types. With these studies in mind, investigations are necessary to elucidate the interaction between soil moisture and soil texture, where larval populations are monitored under different common soil types in the field, under a range of soil moisture treatments. Future work should also include extreme climate events, such as drought and flooding, as the frequency of such events are predicted to increase in the future (Pachauri et al., 2014). This way, we can gain a better picture of how belowground scarab pest status will change in the future.

Several studies have reported responses from other scarabs to soil moisture. For example, within the genus *Cyclocephala*, larvae are significantly more abundant and also have higher mass in irrigated, compared to non-irrigated plots (Potter et al., 1996). Survival of dusky pasture scarab larvae have been shown to be optimal between -100 and -150 kPa, while in saturated soils, larval survival is negatively proportional to the length of exposure (Davidson et al., 1972b). While studies involving *R. majalis*, have shown that larvae move quickly toward the surface when the moisture content of the soil is increased, yet little movement is exhibited in response to drought conditions (Villani and Wright, 1988).

Changes in soil moisture will also impact the host plants of scarab larvae. In addition to this, the diffusion of plant root volatiles is reduced in high soil moisture, however, some moisture is required to prevent total vertical diffusion (Hiltpold and Turlings, 2008). Indeed, natural enemies of scarab larvae, such as entomopathogenic nematodes (**Table 1**) (EPNs), are more effectively recruited by plant volatiles and have higher virulence in soils with high moisture content (Grant and Villani, 2003). Therefore future studies into the effects of different soil moisture contents within a variety of soil types, would also benefit to consider how the natural enemies and pathogens respond under these conditions. This way a more holistic and ecologically relevant picture can be constructed.

### Fertilization

The response of soil dwelling root-feeders to fertilization has received some attention within the literature. Frew et al. (2013) found that the application of nitrogen, phosphorus, and potassium (NPK) fertilizers promoted more nutritionally superior grass species, which in turn increased abundance of dusky pasture scarab larvae. However, Potter et al. (1996) who investigated the effects of different agricultural practices on scarab populations over 3 years and found no significant effect of NPK fertilizer on *Cyclocephala* spp. density or growth. On the other hand, Radcliffe (1970) added organic (cow dung) fertilizer to the soil and found that this lessened the damage to grass roots by *C. zealandica.* This may have been where the larvae switched from feeding on the grass roots to the increased provision of organic matter in the soil, or the addition of excess organic matter may have contributed to better compensatory root growth in response to damage, or a combination of both. In the same study it was found that larvae development was more advanced when treated with nitrogen fertilizer (Radcliffe, 1970). It has also been shown that the addition of organic fertilizer increases the mass gain of *C. zealandica* larvae (Wightman, 1974). In contrast to these findings, other studies on *C. zealandica* have shown the addition of nitrogen fertilizers has had no effect on larval feeding and survival (Prestidge et al., 1985) or population density (Prestidge and East, 1984), with similar responses found with *Popillia japonica* Newman (subfamily: Rutelinae) to the application of NPK fertilizer (Crutchfield et al., 1995). Other root feeding insects have been shown to respond positively to the addition of nitrogen fertilizer, such as the rice weevil larvae [*Lissorhoptrus oryzophilus* Kuschel (Curculionidae, Erirhininae)] and the western corn rootworm larvae [*Diabrotica virgifera virgifera* LeConte (Chrysomelidae, Galerucinae)] (Spike and Tollefson, 1988). In the comprehensive review of belowground herbivores by Brown and Gange (1990), it was suggested that the timing of fertilization is important to the effect on the root feeding larvae. They suggested that if nitrogen fertilizer is applied before larvae are present then this promotes root growth, which in turn gives a greater food supply to larvae, while if fertilizer is added after larval establishment then the damage to grasses is less (Spike and Tollefson, 1988).

It is known in some plants that when nitrogen is limiting in the soil, plant defense investment increases in the leaves (Schmelz et al., 2003; Chen et al., 2008). Low soil nitrogen content could similarly affect root defense investment allocation, thereby impacting the root-feeding scarab beetle larvae populations. It has been suggested that fertilization may cause a reduction in the defensive root compounds (Hol, 2011; Erb and Lu, 2013). These may be direct secondary defenses affecting scarab feeding or performance, or indirect defenses involving recruitment of natural enemies such as EPNs (see section on ‘Pathogens, natural enemies and symbionts’ below). Such plant responses to fertilization addition could be linked to arbuscular mycorrhizal fungal (AMF) associations. AMF associations have been shown to increase induced plant defense responses (Pozo and Azcón-Aguilar, 2007), but root colonization by AMF is known to be reduced when soil nutrients (particularly nitrogen and phosphorus) are high (Vannette and Hunter, 2009; Smith and Read, 2010). Therefore any decrease in plant defenses in response to high nitrogen, could be mediated by limited AMF colonization.

Overall, the literature is not consistent regarding the impact of fertilization on scarab larvae and similar species, although both positive and null effects seem to be the most common responses reported. Any positive effect is likely to be due to an increase in organic matter for younger instar scarabs to ingest and an increase in the nutritional value of host plant species. An increase in nutrient availability may also result in an increase in the tolerance of the host plant to herbivory, although this is likely to be dependent on the nutrient and specific herbivore in question (Wise and Abrahamson, 2007). This may also impact on important microbial plant associations in the soil (Smith and Read, 2010), which can indirectly impact on herbivores (Bennett and Bever, 2007; Biere and Bennett, 2013). Therefore soil fertility may promote root-feeding scarabs, but also may increase plant tolerance to herbivory as well as benefit the natural enemies of scarabs belowground. Continued research should aim to include as many contributing factors to plant–insect interactions within the soil (such as AMF and EPNs) as possible, as these are likely to produce outcomes more relevant in the field.

## Biotic Soil Factors

### Pathogens, Natural Enemies and Symbionts

Scarabs have a number of natural enemies and insect pathogens that threaten their survival. Scarab larvae have evolved within the soil environment, which naturally brings them in close contact with numerous soil organisms and microbiota, some of which are pathogens (Jackson and Klein, 2006). Here we discuss some pathogens and natural enemies that have been identified to hold potential as biocontrol agents against scarab larval pests in the field.

Entomopathogenic fungi are ubiquitous in soils, particularly those within the genera *Metarhizium* and *Beauveria.* Greyback cane beetle larvae are easily infected by the entomopathogenic fungus (**Table 1**) *M. anisopliae.* The impact of this naturally occurring fungus on the larval populations is not density dependent and as such has been shown to account for a fixed mortality rate, regardless of the population density, while the spores are known to be resistant to many agricultural practices (Sallam et al., 2003, 2007). This fungus has been isolated and commercialized as BioCane^TM^ and used as a fungal biocontrol that in trials has shown more than 50% control of the canegrub after 6 months of a single application (Logan et al., 2000). Interestingly Berón and Diaz (2005) carried out susceptibility trials of the Argentine scarab larvae to different strains of *M. anisopliae*. All strains showed low virulence against the larvae, possibly due to the lack of host specificity to the Argentine scarab. However, a particular strain of the entomopathogenic fungus *Beauveria bassiana*, did show up to 70% mortality in Argentine scarab larvae. The differences in virulence of *M. anisopliae* toward different scarab larvae species shows how the insect response to microbial pathogens can often be species specific, and can vary significantly. Another *Beauveria* sp. that has shown success as a biocontrol is *B. brongniartii*, which has been successful acting against a broad range of hosts. Some native strains have been isolated from *Melolontha melolontha* Linneaus (subfamily: Melolonthinae) and used as pest controls across Europe with good success (Dolci et al., 2006). Similar work with *Beauveria* strains isolated from Madagascar and Turkey have also seen success (Maurer et al., 1997; Sevim et al., 2010). These are further examples of successful isolation and application of naturally occurring scarab pathogens.

A significant pathogenic microorganism, particularly noted in efficacy against the greyback cane beetle larvae, is the protozoan *Adelina* sp. which is a density dependent pathogen (Robertson et al., 1998). High *Adelina* incidence causes a drop in the larval population which in turn impacts on the *Adelina* incidence in the soil. Interestingly Sallam et al. (2003) found that *Adelina* incidence was higher in soil with grass cover compared to bare soil areas, which could be due to higher moisture retention and cooler temperatures. Responses such as these should be taken into account when managing larval populations in agriculture to optimize natural pathogen efficacy.

Within New Zealand, the bacteria *Serratia entomophila* and *S. proteamaculans* were isolated from *C. zealandica* as the cause of amber disease, which leads to the cessation of feeding of the scarab grub resulting in eventual death (Hurst et al., 2004). These bacteria were developed as biopesticides against scarabs and have been used for almost 20 years as biocontrol agents. These are further examples of microbial pathogens adapted to their host, and their host range which were used to great success as a control method of scarabs (Hurst et al., 2000).

There are a number of viruses that infect scarabs, such as pox viruses and iridescent viruses; however, little research has been done on their potential as biocontrols, and their presence and effect on scarab populations under natural conditions has not yet been documented (Jackson and Glare, 1992). Damage by the Dynastinae scarab larvae within the genus *Oryctes* has been successfully mitigated via the *Oryctes* virus (Huger, 2005), which is a unique virus, in that it was identified as the first rod-shaped, non-occluded insect virus, and is highly infectious. It has been isolated, purified and used in pest control for over 10 years, but it has low success on any species outside of the target scarab genus *Oryctes* (Huger, 2005). Current research is focused on selecting strains of the virus for greatest persistence in the environment.

One of the major natural enemies of scarabs are EPNs, which are internal parasites of scarabs. They do not act alone, but rather it is their association with entomopathogenic bacteria that kill the scarab hosts. *Steinernema* and *Heterorhabditis* are the two genera of EPNs and there are a number of species within both genera that infect scarabs (Klein, 1993). The EPNs kill the larvae via their symbiotic bacteria *Xenorhabdus* sp. Several species have been isolated from scarab grubs, such as *Steinernema glaseri*, *S. anomaly*, *Heterorhabditis megidis*, and several different strains of *S. carpocapsae* and *H. bacteriophora* (Klein, 1993), their potential to control scarab larvae populations is being investigated. Some nematodes have shown success in laboratory and field trials against scarab larvae, with particular interest in *S. scarabaei* as an effective control against a range of scarabs dominant in North America and Asia (Stock and Koppenhöfer, 2003). However, other efforts to use EPNs in the field have not been successful, which have been attributed to a lack of understanding of the nematode–bacterium complex and differences in target species susceptibility, biology or behavior (Klein, 1993; Georgis et al., 2006). Recently Wu et al. (2014) tested and compared the virulence of four EPN species and their interactive effects with entomopathogenic fungi against the scarab larvae of *Cyclocephala lurida* Bland (subfamily: Dynastinae). They concluded that the impact of *H. bacteriophora* alone or in combination with the fungal pathogens was comparable to that of an imidacloprid insecticide against the larvae. This indicates the potential EPNs have as biocontrols and that further work is warranted to fully elucidate the interaction between natural enemies, pathogens, and host. Plants can recruit EPNs via attractive volatile signals as a natural defense strategy (Grewal et al., 1994; Rasmann et al., 2005). It has been shown that EPNs can be selectively bred for enhanced responsiveness to these volatile cues (Hiltpold et al., 2010), meaning that improved efficacy of the commercial EPN use is still ongoing and holds great potential as a biological control method of scarabs in agriculture and industry.

Finally, diverse communities of endosymbiotic bacteria (**Table 1**) that assist with the digestion of plant material, particularly cellulose and hemicelluloses, live within the hindguts of scarab larvae (Cazemier et al., 2003; Huang et al., 2010). Pittman et al. (2008b) found that there were species within the bacterial community of the greyback cane beetle larvae hindgut that were consistently found within the larvae across their geographical distribution. These bacteria were successfully transformed and reintroduced into the hindgut of the larvae, which indicates they are strong candidates to control the populations of greyback cane beetle larvae through the expression of anti-feeding compounds within the larval gut (Pittman et al., 2008a). Non-resident bacteria are normally not useful in such paratransgenic control methods because they are unable to remain established within the gut (Chapco and Kellin, 1994). Therefore the discovery, successful transformation and establishment of these candidate bacteria within the greyback cane beetle larval gut provides good grounding for the future development of paratransgenic control methods of the larvae.

## Applied Perspectives

**FIGURE 5:**
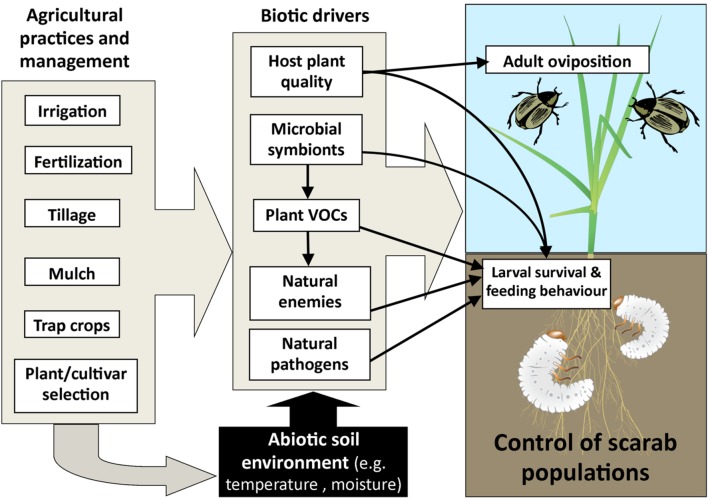
**Diagram of agricultural practices and management factors that impact on plant and soil factors (abiotic and biotic), which in turn can influence oviposition by adults together with larval survival and feeding behavior.** Arrows indicate key linkages between interacting factors.

We have discussed the impacts of some abiotic and biotic factors within the soil environment that impact on scarab larval populations. Many agricultural practices interact with these factors within the soil, and could potentially mitigate or exacerbate scarab damage to grasses and crops (Barnett and Johnson, 2013; see **Figure 5** for a summary of key interactions within an applied context).

Scarab larvae have been shown to respond to the application of fertilizers (Wightman, 1974; Frew et al., 2013). However, it is important to note that AMF plant associations can be negatively impacted by fertilization (Smith and Read, 2010). Therefore, the application of NPK fertilizer, particularly to newly establishing crops or pastures should be kept to a minimum, to minimize any positive impacts on scarab populations and to ensure effective AMF colonization to enhance grass productivity and defenses. The addition of mulch, is commonly used to conserve moisture and generally improve soil fertility, and therefore could reduce the priming of plant defenses to herbivores by reducing AMF colonization (Grant et al., 2005; Smith and Read, 2010).

Mulch also affects temperature, which in turn may influence scarab beetle larvae. Different types of mulch have been shown to have different effects on the temperature of the soil (Ramakrishna et al., 2006). For example, polythene mulch has been shown to increase soil temperature by 6°C, while straw mulch also increased soil temperature, but to a lesser extent (Ramakrishna et al., 2006). Contrastingly, a study by Lal (1974) found that mulch consistently decreased the maximum soil temperature across a range of depths (5, 10, and 20 cm), with the biggest difference of 8°C, seen at 5 cm below the soil surface. Tillage is another agricultural practice which has been shown to affect soil temperature (Griffith et al., 1973; Malhi and O’Sullivan, 1990; Licht and Al-Kaisi, 2005). Conventional tillage increases top soil temperatures by 2.8°C compared with no tillage (Malhi and O’Sullivan, 1990), although smaller increases in temperature of 1.9°C have also been reported (Licht and Al-Kaisi, 2005). Higher soil temperatures (depending on climatic conditions) reduce greyback cane beetle populations (Horsfield et al., 2008), and first instar larvae of the dusky pasture scarab have been found to be the most temperature sensitive (Davidson et al., 1972a). However, other common practices such as irrigation are known to lower soil temperatures by up to 3.8°C (Wang et al., 2000).

Taking these effects into account, the timely refrain from irrigation alongside the application of polythene or straw mulch coupled with tillage, for example, could raise soil temperature sufficiently to impact on larval populations. However, limiting soil moisture could decrease the efficacy of EPN populations within the soil at controlling scarab populations. The effects of raising temperatures in this manner on crop health and yield, however, should also be investigated.

The effects of other land management practices on scarab larvae populations have been reported such as the study by Potter et al. (1996) who found that intense mowing of grasses and the addition of aluminum sulfate treatments significantly decreased populations of *Cyclocephala* spp., as well as the average larval mass. This study, however, only was done within one soil type, which is a critical factor (Cherry and Allsopp, 1991; Matthiessen, 1999), and scarab responses may differ under different soils.

Many crops have irrigation systems in place to ensure sufficient water is supplied, which can lead to very different soil conditions compared to natural systems. Mulch, as discussed, is commonly used in agriculture to conserve moisture and increase fertility of soil, and so it naturally follows that in mulched systems, moisture retention of the soil will be higher (Moody et al., 1963; Lal, 1974; Ramakrishna et al., 2006). Host plant location by larvae beneath the soil surface could be improved under these moist soil conditions due to the fluid dynamics of root exudates (Gouinguené and Turlings, 2002; Hiltpold and Turlings, 2008). However, at the same time, natural enemies such as EPNs will also benefit from this phenomenon as it has been shown across several species that EPN virulence increases with soil moisture content (Kung et al., 1991; Grant and Villani, 2003; Frew et al., 2013). Therefore, as practices such as fertilization may decrease EPN attracting volatiles while irrigation enhances EPN mobility and survival, effective strains of host specific EPNs should be applied to pastures or crops requiring little fertilization alongside ample irrigation to effectively repress scarab larval populations.

Other soil antagonists can be impacted by land use practices. For example, larvae of the scarab *Ataenius spretulus* Haldeman (subfamily: Aphodiinae) were found, within a golf course environment, to be in greater abundance where the turf had been mowed to fairway height (1.6 cm), compared with turf mowed to rough height (5.1 cm). This correlated with the number of larvae found to be infected with a bacterial pathogen, *Bacillus* sp., where 68% of larvae were infected in the turf mowed to rough height, compared to 34% of larvae infected in turf mowed to fairway height. In addition to this, *Anoplognathus* spp. and *Sericesthis* spp. larval populations have been shown to peak under moderate grazing pressure, yet were lowest under high intensity grazing (Roberts and Morton, 1985). These findings alone are unlikely to have a direct applied significance to all scarab larval pest management. However, they may provide critical information for other managed grassland systems, where decreasing regular mowing or allowing high intensity grazing may mitigate larval infestations in future years. Common practices as mowing should be investigated for their impacts on critical soil abiotic factors such as moisture alongside scarab larval populations and their interactions with natural pathogens.

In direct attempts to mitigate damage caused by insect herbivores, the ‘push–pull’ system is a method which aims to utilize repellant or unattractive plants while simultaneously using attractive yet less valuable plants to attract pests away from valuable crops or pastures (Pickett et al., 2014). A similar system could be utilized against scarab larval pests. For example, where African black beetle populations are problematic, the use of *T. repens* and *N. lolii* infected *L. perenne* could be used as a repellant [the former of which may also be effective against Christmas beetle larvae (Davidson and Roberts, 1968a)], while *L. perenne* and *P. dilatatum* could be utilized within ‘trap crops,’ particularly as areas with *P. dilatatum* are also preferred sites for oviposition. Indeed, *P. dilatatum* could also be useful, alongside *C. dactylon* in ‘trap cultures’ for other Dynastinae species such as the Argentine scarab (Carne, 1957a). It has been suggested, however, that the efficacy of ‘push–pull’ systems would be improved if a better understanding of the mechanisms were obtained, for example the specificity and distance ranges of plant volatile cues (Eigenbrode et al., 2016).

In the end, where effective biocontrol methods are commercially available, these should be employed in conjunction with the use of agricultural and land-use practices, such as irrigation and mowing (where applicable) to create optimal conditions for efficacy and infectivity. Where scarab plant host preferences are known (for feeding or oviposition), these can be employed in ‘push–pull’ strategies, to limit larval populations in areas of interest. Where either of these are unavailable or remain unknown, such is the case for some of our focal species, timely utilization of certain land-use practices can be applied to create poor conditions for the scarab populations (e.g., during the first instar, when larvae are most vulnerable to temperature stress). Indeed, in either situation, encouragement of natural beneficial soil microbes (such as AMF) should also be applied. However, as there are gaps in the knowledge for ecology of many scarab species, the direction of future research is of primary importance in improving strategies to limit pest scarab larvae in grasses across Australasia.

## Directions for Future Research

### Basic Ecology

Some of the work on the basic ecology of scarab larval pests to grasses was carried out over 20 years ago (Carne, 1957a; Carne and Chinnick, 1957; Ridsdill-Smith, 1975), with little research on particular species since. It is our belief that for those species where there remains some paucity of knowledge in their basic ecology, feeding trials looking at host preference alongside population monitoring under different conditions (this includes monitoring of abiotic factors and microbial sampling) should be prioritized. With this knowledge, more effective implementation of strategies such as ‘push–pull’ systems or other agricultural practices that suppress scarab beetle populations can be applied within context. This means management systems could take into account species specific responses, accounting for local abiotic and biotic interactions.

### Volatile Cues

The effectiveness of classic pest management strategies such as ‘push–pull’ systems have recently been criticized, particularly for focusing too much on long-range effects, and should consider all cues that can work synergistically (Eigenbrode et al., 2016). Indeed we would concur with this framework for application to belowground pests, but such behavioral cues would first require investigation. We recommend that future research should investigate olfactory cues of pest larvae and their natural enemies belowground to plant roots, and how these may interact with common agricultural and land-use practices. Experiments such as those carried out by Rasmann et al. (2005) using six-arm olfactometers are an ideal starting point to determine attractiveness of plant species to scarab larval pests and/or their natural enemies.

### Pathogens and Microbes

Biocontrol of scarab pests has been particularly successful where a naturally occurring pathogen is identified, isolated and then applied within its naturally occurring range (Maurer et al., 1997; Hurst et al., 2000; Sallam et al., 2003, 2007; Dolci et al., 2006; Sevim et al., 2010). Hence, knowledge of belowground community composition is important if native microbes or EPNs are to be utilized in the control of insect pests in the soil. Using methods similar to that of Sevim et al. (2010), the presence of naturally occurring scarab pathogens could be identified using a baiting method (Zimmermann, 1986). The pathogen can then be isolated from infected larvae and the DNA sequenced; effective isolates can then be used in bioassays to test pathogenicity against the target pest species. We recommend the isolation, identification and ultimately the application of natural pathogens, where possible. The persistence of scarab pathogens in the soil indicates some level of evolutionary success, which should be exploited in efforts to control problematic species.

## Concluding Remarks

Here, we have presented information on several key scarab larval species within three subfamilies, known to cause significant damage to grasslands and crops within Australia and New Zealand. While the ecology of some species has been well researched, information on others, including the Argentine scarab, has not been described in any detail. The feeding behavior and general ecology has been investigated for species such as African black beetle larvae and greyback cane beetle larvae. These pests have had significant attention as a result of their impact on agriculture, and control methods such as the application of natural pathogens, or the application of host plant endophytes have shown noteworthy promise. Although our knowledge is somewhat limited for some species, there is good evidence that changes in management can potentially have a large impact in limiting damage to crops and grasslands. Overall it seems clear that, in terms of improved pest management of scarab larvae, it does not make sense to run before we can walk. Immediate research concerns should lie with filling knowledge gaps in the ecology of scarab species within Australasia. This should include assessing population dynamics, interactions and influences with abiotic factors within the local environment. In addition to this, successful biocontrol strategies, both within and outside Australasia, have utilized naturally occurring pathogens and natural enemies, which are adapted to their host and local environment. Therefore, similar strategies need to be central to future biocontrol research on Australasian scarab pests. This will necessitate multi-factorial studies to investigate how best to integrate these antagonists under different abiotic conditions. Overall, pest management strategies that are applied within context would be more effective with an improved fundamental ecological understanding of key scarab pests.

## Author Contributions

AF wrote the main body of the review, contributing the majority of the intellectual content and concept of the review. KB read the review in detail, giving advice and contributing important intellectual content. KB was responsible for the production of **Figure 3** MR read the review in detail, giving advice and contributing intellectual content. UN read the review in detail, giving advice and contributing intellectual content. SJ aided in the concept of the review, contributing important intellectual content.

## Conflict of Interest Statement

The authors declare that the research was conducted in the absence of any commercial or financial relationships that could be construed as a potential conflict of interest.
